# An Internet-Based Counseling Intervention With Email Reminders that Promotes Self-Care in Adults With Chronic Heart Failure: Randomized Controlled Trial Protocol

**DOI:** 10.2196/resprot.2957

**Published:** 2014-01-30

**Authors:** Robert P Nolan, Ada YM Payne, Heather Ross, Michel White, Bianca D'Antono, Sammy Chan, Susan I Barr, Femida Gwadry-Sridhar, Anil Nigam, Sylvie Perreault, Michael Farkouh, Michael McDonald, Jack Goodman, Scott Thomas, Shelley Zieroth, Debra Isaac, Paul Oh, Miroslaw Rajda, Maggie Chen, Gunther Eysenbach, Sam Liu, Ahmad Zbib

**Affiliations:** ^1^Behavioral Cardiology Research UnitUniversity Health NetworkToronto, ONCanada; ^2^Faculty of MedicineUniversity of TorontoToronto, ONCanada; ^3^Division of CardiologyPeter Munk Cardiac CenterUniversity Health NetworkToronto, ONCanada; ^4^Centre de RechercheInstitut de Cardiologie de MontréalMontréal, QCCanada; ^5^Département de PsychologieUniversité de MontréalMontréal, QCCanada; ^6^Faculty of MedicineUniversity of British ColumbiaVancouver, BCCanada; ^7^Department of CardiologySt. Paul's HospitalVancouver, BCCanada; ^8^Department of Food, Nutrition, and HealthUniversity of British ColumbiaVancouver, BCCanada; ^9^Department of Medical Health InformaticsLawson Health Research InstituteLondon, ONCanada; ^10^Faculté de PharmacieUniversité de MontréalMontréal, QCCanada; ^11^Division of CardiologyMount Sinai HospitalToronto, ONCanada; ^12^Faculty of Kinesiology and Physical EducationUniversity of TorontoToronto, ONCanada; ^13^Faculty of MedicineUniversity of ManitobaWinnipeg, MBCanada; ^14^Cardiac Transplant ClinicLibin Cardiovascular Institute of AlbertaCalgary, ABCanada; ^15^Department of Cardiac RehabilitationToronto Rehabilitation InstituteToronto, ONCanada; ^16^Division of CardiologyQueen Elizabeth II Health Sciences CenterHalifax, NSCanada; ^17^Dalla Lana School of Public HealthUniversity of TorontoToronto, ONCanada; ^18^Centre for Global eHealth InnovationUniversity Health NetworkToronto, ONCanada; ^19^Consumer eHealthHeart and Stroke Foundation of CanadaToronto, ONCanada

**Keywords:** e-counseling, chronic heart failure, lifestyle intervention, Internet-based intervention, quality of life

## Abstract

**Background:**

Chronic heart failure (CHF) is a public health priority. Its age-standardized prevalence has increased over the past decade. A major challenge for the management of CHF is to promote long-term adherence to self-care behaviors without overtaxing available health care resources. Counseling by multidisciplinary health care teams helps to improve adherence to self-care behaviors and to reduce the rate of death and hospitalization. In the absence of intervention, adherence to self-care is below recommended standards.

**Objective:**

This trial aims to establish and evaluate a Canadian e-platform that will provide a core, standardized protocol of behavioral counseling and education to facilitate long-term adherence to self-care among patients with CHF.

**Methods:**

Canadian e-Platform to Promote Behavioral Self-Management in Chronic Heart Failure (CHF-CePPORT) is a multi-site, double blind, randomized controlled trial with a 2 parallel-group (e-Counseling + Usual Care vs e-Info Control + Usual Care) by 3 assessments (baseline, 4-, and 12-month) design. We will identify subjects with New York Heart Association Class II or III systolic heart failure from collaborating CHF clinics and then recruit them (n=278) by phone. Subjects will be randomized in blocks within each site (Toronto, Montreal, and Vancouver). The primary outcome will be improved quality of life, defined as an increased number of subjects with an improvement of ≥5 points on the summary score of the Kansas City Cardiomyopathy Questionnaire. We will also assess the following secondary outcomes: (1) diet habits, depression, anxiety, smoking history, stress level, and readiness for change using self-report questionnaires, (2) physical activity level, current smoking status, and vagal-heart rate modulation by physiological tests, and (3) exercise capacity, prognostic indicators of cardiovascular functioning, and medication adherence through medical chart review. The primary outcome will be analyzed using generalized estimation equations with repeated measures on an intention-to-treat basis. Secondary outcomes will be analyzed using repeated-measures linear mixed models with a random effects intercept. All significant main effects or interactions in the statistical models will be followed up with post hoc contrasts using a Bonferroni correction with a 2-sided statistical significance criterion of *P*<.05.

**Results:**

This 3.5-year, proof-of-principle trial will establish the e-infrastructure for a pan-Canadian e-platform for CHF that is comprised of a standardized, evidence-based protocol of e-Counseling.

**Conclusions:**

CHF-CePPORT is designed to improve long-term adherence to self-care behaviors and quality of life among patients with CHF. It will demonstrate a distinct Canadian initiative to build capacity for preventive eHealth services for patients with CHF.

**Trial Registration:**

ClinicalTrials.gov NCT01864369; http://clinicaltrials.gov/ct2/show/NCT01864369 (Archived by WebCite at http://www.webcitation.org/6Iiv6so7E).

## Introduction

### Chronic Heart Failure Syndrome

Chronic heart failure (CHF) is a progressive clinical syndrome in which the heart is unable to pump oxygenated blood sufficiently to meet metabolic demands during exercise or at rest [1]. It is a major cause of hospitalization and mortality, and it is the only major cardiovascular disease that is increasing in prevalence [2]. For example, in Canada, the age-standardized prevalence of CHF has risen from 1585 to 2510 cases per 100,000 over the past decade [3]. The 1-year hospital readmission rate is 40% [4], and the mortality rate after the first year of a CHF diagnosis is 25%-40%. Patients with CHF experience many symptoms such as shortness of breath and fatigue [5]. Self-care behaviors are critical to symptom management and quality of life. These behaviors include maintaining a healthy diet that is low in fat and sodium, limiting alcohol and fluid intake, maintaining a healthy body weight, exercising regularly, reducing stress, and smoke-free living [6].

A key challenge is to improve quality of life and long-term adherence to self-care behaviors for patients with CHF without overtaxing health care resources. In the absence of intervention, adherence to self-care is problematic with regard to medications (50%-96%), physical activity (9%-53%), dietary restriction of sodium (20%-71%), and daily monitoring of weight (20%-80%) [7]. Meta-analysis has shown that multidisciplinary counseling to promote self-care following hospital discharge for CHF reduces mortality (relative risk-RR=0.75, 95% CI 0.59-0.96), CHF-related hospitalizations (RR=0.74, 95% CI 0.63-0.87), and all-cause hospitalizations (RR=0.81, 95% CI 0.71-0.92) [8].

### e-Counseling for Patients

We support the conclusions from recent meta-analytic reviews that call for further development of an e-Counseling strategy for patients with cardiovascular conditions, including CHF [8-10]. An e-Counseling strategy may be well suited to reinforce long-term adherence to self-care among patients with CHF, and in turn improve quality of life while reducing the high rate of hospitalization and mortality. The feasibility of this approach is underscored by the observation that 80.3% of Canadians reported having personal access to the Internet in 2010, including 70%-76% in the two lowest income quartiles, 80% who were between 45-64 years old, and 51% between 65-74 years old [11]. Moreover, we surveyed 100 patients with CHF about whether “It was easy for [them] to get access to a computer at home” (1=Strongly disagree, 5=Strongly agree) [12]. The mean response was 4.4, SD=1.1. In keeping with recent studies [13,14], these data indicate that e-Counseling is very likely to be used by patients with CHF in our clinics.

The primary objective of this trial is to establish and evaluate a Canadian e-platform for e-Counseling and education to enhance quality of life and to facilitate long-term adherence to self-care among patients with CHF. This proof-of-principle trial builds upon: (1) previous clinical trials in e-Counseling, telehealth, and telemonitoring, as well as observational studies by our team [12,15-28], and (2) our contributions to Canadian consensus guidelines for the clinical management of CHF [29-31]. This trial will be undertaken in collaboration with the Consumer eHealth platform of the Heart and Stroke Foundation of Canada. Findings from the trial will help extend access to e-Counseling, interactive e-tools, and self-help information for patients with CHF.

## Methods

### Procedures and Features

Procedures and features of our methods have been reviewed according to the Consolidated Standards of Reporting Trials (CONSORT) standards for clinical trials in eHealth [32,33]. It is reported here in accordance with the CONSORT-EHealth (v.1.6.1) checklist [34].

### Trial Design

**Figure 1:**
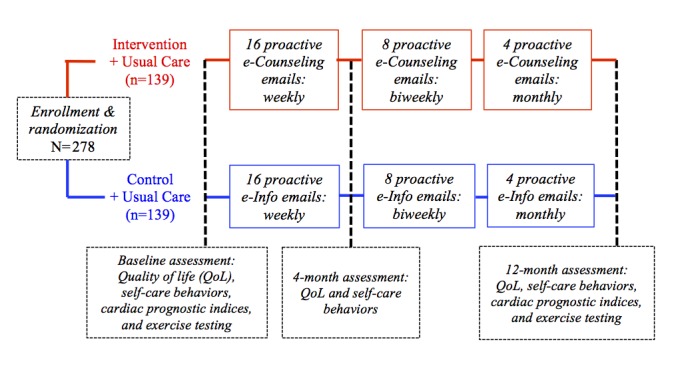
Schematic summary of the trial design.

The Canadian e-Platform to Promote Behavioral Self-Management in Chronic Heart Failure (CHF-CePPORT) is a multi-site, double blind, randomized controlled trial (NCT01864369) with 2 parallel-groups (e-Counseling + Usual Care vs e-Info + Usual Care) and 3 assessment periods (baseline, 4-, and 12-month). Figure 1 shows the research design. This trial was modeled after 2 randomized controlled trials on telehealth and eHealth completed by our team [19,22,23], and after 2 exemplary trials of telehealth that are recently completed [35,36] or in progress [37].

### Hypotheses

#### Primary Hypothesis

Our primary hypothesis is that more subjects in the e-Counseling + Usual Care arm will experience significant improvement in the quality of life at the 12-month assessment than in the Control arm. This improvement will be defined as a clinically meaningful increase of ≥5 points [38] on the summary score of the Kansas City Cardiomyopathy Questionnaire (KCCQ) [39] from baseline.

#### Secondary Hypotheses

Our secondary hypotheses are that the e-Counseling + Usual Care arm will be associated with significant improvement in the quality of life, adherence to self-care, and psychological adjustment from baseline to 4- and 12-month assessments. These outcomes will be measured using the following objective and validated indices: (1) the KCCQ (≥5 points increase in the summary score at the 4-month assessment) for quality of life, (2) the Canadian version of the Diet History Questionnaire II (DHQ II) [40] for diet habits, (3) the mean 4-day step count (as measured by accelerometry) [41,42] and the Physical Activity Scale for the Elderly (PASE) questionnaire [43] for physical activity level, (4) the medication possession ratio (MPR) [20] for medication adherence, (5) the generalized anxiety disorder (GAD-7) scale [44] and Patient Health Questionnaire (PHQ-9) [45] for anxiety and depressive symptoms, respectively, (6) the Perceived Stress Scale (PSS) [46] for stress level, and (7) Prochaska’s algorithm [47] for readiness to change self-care behaviors.

In addition, we hypothesize that the e-Counseling + usual care group will perform better than the Control groups at the 12-month assessment on the following prognostic measures of cardiovascular functioning: (1) increased exercise capacity as measured by the 6-minute walk test (6MWT) [48], (2) increased vagal-heart rate modulation [44,45] as measured by electrocardiography (ECG), (3) decreased values of the Seattle Heart Failure Model (SHFM) [49-51], N-terminal pro-brain natriuretic peptide (NT-proBNP) [52,53], high sensitivity C-reactive protein (hsCRP) [52,54], and (4) increased VO_2_ peak [55] during cardiopulmonary exercise testing.

### Participant Inclusion/Exclusion Criteria

Patients with CHF will be eligible for the trial if they meet all of the following criteria: (1) male and female patients 18 years or older who are diagnosed with heart failure with reduced ejection fraction (systolic HF) that corresponds to New York Heart Association (NYHA) Class II or III for 3 or more months prior to enrollment, (2) documentation of left ventricular ejection fraction (LVEF) ≤40%, (3) if the patient has been stable throughout 12 months prior to enrollment, documentation will confirm impairment of LVEF by contrast ventriculography, radionuclide ventriculography, or quantitative echocardiography, (4) no worsening of CHF for 1 month prior to recruitment, as determined by the referring cardiologist, (5) confirmation by referring cardiologist that medical treatment includes an optimal and stable dose of angiotensin converting enzyme inhibitor, beta-blocker, and aldosterone antagonist where indicated for at least 1 month prior to enrollment in this study (Patients not treated with a beta-blocker will be enrolled if this was previously prescribed, but not tolerated by them. Use of digitalis or diuretic is optional), (6) subject is not *currently* enrolled in a formal exercise program, (7) comprehension of English or French, (8) subject is familiar with the use of and has access to a personal computer, email, and the Internet, and (9) subject provides informed written consent.

Patients with CHF will be ineligible if they fit with any of the following exclusion criteria: (1) documentation at enrollment of renal failure (serum-creatinine ≥300 micromoles/L, ≥3.0 mg/dL), significant liver disease (alanine transaminase >3-fold upper limit of normal), or poorly controlled diabetes mellitus (fasting blood glucose >10 mmol/L or hemoglobin A1c >8%, or current diagnosis of autonomic neuropathy, ketoacidosis, or hyperosmolar state), (2) current symptomatic hypotension and/or systolic blood pressure ≤85 mmHg, (3) persistent systolic or diastolic hypertension (systolic >170 mmHg or diastolic >100 mmHg despite antihypertensive therapy), (4) CHF secondary to any of the following conditions–primary uncorrected valvular cardiomyopathy, predominant right-sided heart failure, or noncardiac disease (eg, pericardial disease, complex congenital heart disease), (5) cardiovascular comorbidities/procedures that include stroke, acute myocardial infarction, or planned cardiac surgery within 4 weeks before enrollment, (6) severe obstructive, restrictive, or other chronic pulmonary disease, (7) previous heart transplant or is on a wait list for heart transplant at the time of enrollment, (8) diagnosis of major psychiatric disorder (eg, psychosis) or drug/alcohol abuse in past year, and (9) diagnosis of noncardiac disease (eg, cancer) that is likely to shorten life expectancy to <2 years.

### Recruitment Procedure

Cardiologists, who are coinvestigators in this trial, will identify patients with CHF in their care who meet our recruitment criteria. A clinic staff member will introduce the study to the potential subjects and solicit their verbal consent to be contacted by our research team. The contact information of consented individuals will be sent via secure fax to the Behavioral Cardiology Research Unit at University Health Network (UHN), Toronto, Canada. Individuals will be telephoned by our research team to obtain verbal consent to participate and to schedule a baseline assessment appointment to be held at one of the three trial sites (Toronto, Montreal, and Vancouver). We will obtain written informed consent from subjects for trial participation, email communication, and review of medication history at the beginning of their baseline assessment appointment. Once they have completed their baseline assessment, subjects will be randomized into the intervention or control group. We have received ethics approval from our coordination site (UHN). At the time of manuscript submission, we are seeking ethics approval from the Vancouver and Montreal trial sites.

### Intervention

#### Clinical/Theoretical Framework

New taxonomies of techniques for health behavior change have been reported [56,57]. Based on this work, our team has recently published a systematic review of how behavior change techniques are utilized in trials of e-Counseling [58]. Our key finding is that e-Counseling programs that were efficacious used a repertoire of at least six behavior change techniques that can be tailored to (or selected by) the individual user. Further, it is critical to present these techniques in an organized manner, within a framework of preventive counseling that is evidence-based and clinically relevant.

CHF-CePPORT is not “therapy” per se. However, our e-Counseling protocol incorporates key components of two foundational models of behavioral counseling: (1) motivational interviewing (MI) [59], and (2) cognitive-behavioral therapy [60-62]. MI was developed as a set of procedures [63] that build upon educational strategies used in Prochaska’s transtheoretical model [47,64]. The initial goal of MI is to validate the patient’s presenting stage of “readiness” and to tailor feedback or information accordingly. Therapeutic interactions and self-assessment procedures are designed to evoke positive “change talk,” [63] as the patient is directed to identify personally salient goals that are associated with potential change in a targeted behavior. As ambivalence about change is resolved, the patient is directed to “experiment” with behavior change. Goals for change and behavioral feedback are conventionally provided in a manner that is consistent with cognitive-behavioral therapy [60-62]. Strategies to sustain change are collaboratively reviewed to assist the patient in: (1) developing a repertoire of behavioral skills, and (2) building efficacy from performance-based feedback. Meta-analysis shows that in comparison to a standardized intervention or usual care, MI (alone or combined with cognitive-behavioral counseling) is associated with significantly greater reduction in body mass index, total cholesterol, blood pressure, adult smoking, and alcohol abuse, and with increased adherence to diet, exercise, and other “treatment” [65-67].

#### Intervention (e-Counseling + Usual Care) Group

The e-Counseling protocol in CHF-CePPORT builds upon evidence and know-how that we have shown in our previous trials. In the Community Outreach Heart Health and Risk Reduction Trial (COHRT) [23,68,69], we demonstrated that an evidence-based model of telehealth counseling with MI [59] added therapeutic benefit to a recommended standard of preventive counseling at a 6-month follow-up in people with or at elevated risk for cardiovascular disease. In the Internet-Based Strategic Transdisciplinary Approach to Risk Reduction and Treatment (I-START) [19,22,28], our e-Counseling protocol was independently associated with reduced systolic blood pressure, pulse pressure, and total cholesterol (but not with diastolic blood pressure), as well as with increased adherence to psychometrically assessed exercise and diet in people with hypertension. Through our ongoing trial, e-Counseling Promotes Blood Pressure Reduction and Therapeutic Lifestyle Change in Hypertension (REACH) [21], we are establishing whether our e-Counseling protocol independently reduces blood pressure and 10-year absolute risk for cardiovascular disease over a 12-month interval in people with hypertension. The CHF-CePPORT e-Counseling protocol builds upon this evidence [19,22,23,68,69], as well as guidelines from notable trials and reviews of telehealth and eHealth [14,35,37,70-72]. This trial will be a proof-of-principle study for patients with CHF.

The CHF-CePPORT e-Counseling protocol will be delivered in collaboration with the Consumer eHealth platform of the Heart and Stroke Foundation of Canada. It will send 28 emails proactively to each subject in the intervention arm over a 12-month interval (Table 1). Each email will link e-Counseling subjects to a restricted section of our e-platform where they will access multimedia materials and interactive e-tools. As noted above, the clinical method and content of this protocol is consistent with principles of MI. In keeping with I-START [19,22], the e-Counseling messages promote the following: (1) explicit validation of the subject’s stage of “readiness” for behavior change via e-messaging and educational segments, (2) collaborative participation by means of subject-selected menus and explicit messaging to validate the subject’s active participation, and (3) reinforcement of “change talk” [63] through peer modeling, dramatic vignettes, and self-help exercises that are designed to help resolve ambivalence to change. Additionally, MI is most efficacious when combined with other evidence-based counseling methods such as cognitive-behavioral therapy [66]. Accordingly, the e-Counseling protocol will maintain a user-centered approach by working collaboratively with each subject who reports appropriate motivation to change a behavior that they have identified as a priority for change. This includes interactive access to the following therapeutic tools: (1) self-help information and e-tools for self-monitoring self-care behaviors, and (2) developing cognitive-behavioral skills to build and strengthen efficacy [73] to initiate and maintain behavior change. The collaborative tone of the e-Counseling content is consistent with cognitive-behavioral guidelines to reinforce motivation [59,63] and efficacy [73]. Finally, subject engagement in this segment of the e-Counseling program will be reinforced through the use of short films that will complement the e-based self-help information and e-tools. These original short films have been written and produced by our research team, in collaboration with the Heart and Stroke Foundation of Ontario. e-Counseling subjects will continue to receive CHF-related medical care from their health care team during the course of the trial (ie, usual care).

**Table 1:** The 12-month schedule for proactive e-messaging for the CHF-CePPORT trial.

	Month 1-4	Month 5-8	Month 9-12
Schedule for proactive e-messages	Weekly	Biweekly	Monthly
Total # of proactive e-messages	16	8	4

#### Control Group

In addition to usual care, the Control group will be provided with e-messages following the same delivery schedule (Table 1). The e-messages will include brief articles that are randomly selected from the Healthy Living section of the Heart and Stroke Foundation of Canada e-platform. Each e-support article will provide information tailored for a CHF population, such as appointments with physicians and advice about heart healthy guidelines for exercise, diet, smoke-free living, symptom monitoring, and medications. This intervention will be distinct from the e-Counseling group in two ways: (1) information will not be tailored to each subject’s stage of readiness for change, and (2) e-messages will not include e-tools and e-Counseling procedures to increase “readiness” and efficacy to adhere to targeted self-care behaviors.

### Randomization and Blinding

Protection against bias will be accomplished by double blinding. Randomization will be done through a particular website which uses randomly permuted blocks to assign subjects to the e-Counseling group or to the Control group. This process will be conducted in blocks to ensure that group assignment is balanced across our recruitment sites (Toronto, Montreal, and Vancouver) for the overall trial. The individual who will be responsible for randomization at the Behavioral Cardiology Research Unit, UHN has no direct involvement in this trial. In addition, the randomization code will be hidden from trial subjects, as well as all those who will conduct assessments, data processing, and analysis. Thus, the CHF-CePPORT research team members and the trial subjects from the three sites will not be aware of the group assignment of the subjects.

### Outcome Measures

#### Collection Materials in English and French

Data collection materials will be available in both English and French. All materials were developed in English. We will indicate if a published French version of a questionnaire is to be used. If none is available, our Montreal team will translate the English questionnaire into French using a standard back-translation protocol [74].

#### Primary Outcome

The primary outcome is quality of life improvement of subjects at the 12-month assessment, as measured by the number of subjects who demonstrate a clinically meaningful increase of ≥5 points [38] on the summary score of the KCCQ [39]. It is a 23-item questionnaire that assesses the patient’s perception of CHF in terms of physical limitations, symptoms (frequency, severity, and recent change over time), self-efficacy, social interference, and quality of life over the past 2 weeks. The summary score ranges from 0-100, with the lower score reflecting poorer quality of life. Internal consistency is high for all domains (Cronbach alphas=.78-.95), except the 2-item self-efficacy scale (Cronbach alpha=.62). We will employ a validated French-Canadian version [39] of KCCQ in this trial.

#### Secondary Outcomes

The quality of life at the 4-month interval will be assessed by determining the number of subjects who demonstrate a ≥5-point improvement of the KCCQ total score [38,39].

The subjects’ increase in adherence to the recommended intake of vegetables, fruit, dairy, and dietary fat will be evaluated at the 4- and 12-month assessments using the Canadian version [40] of the DHQ II [75], which is also available in French.

The physical activity level of the subjects will be measured in two ways: (1) mean step count, and (2) self-report questionnaire. We will ask subjects to document their daily step count for 7 days prior to their assessments at baseline, 4-, and 12 months. The step count will be measured using an accelerometer (LifeSource/A&D XL-18CN Activity Monitor) that we will provide to each subject. We will calculate the mean 4-day step count using data from the three weekdays and one weekend day that have the highest step count out of the 7-day record [76,77]. Adherence to physical activity over the past week will be measured by the PASE [78], which has been validated in persons with CHF [79]. We will use a French version that was translated by its publisher.

We will confirm the smoking status of those who self-report as a current smoker at the baseline, 4-, and 12-month assessments using salivary cotinine. The current smokers will be defined by having salivary cotinine level ≥10 ng/ml. These subjects will also be screened for the use of nicotine replacement therapy, which can confound their salivary cotinine result. Smoking history will be evaluated using questions from the Survey on Living with Chronic Disease in Canada [80] (ie, “Have you smoked at least 100 cigarettes in your life?”). The French version [80] of the smoking history questions will be also used in this trial.

We will estimate each subject’s medication adherence using MPR [81]. It calculates the cumulative medication supply for *x* days, divided by the total days to the next reﬁll or end of the observation period. MPR has been validated in a previous trial [20]. We will measure MPR with pharmacy refill data for a 4-month period that precedes each of the three assessments.

Anxiety will be measured using the GAD-7 scale [44]. Depressive symptoms will be assessed using the total score from the PHQ-9 [45]. Both questionnaires have been used extensively in health research and have been well validated, including in people with CHF [44,82]. French versions of the GAD-7 and PHQ-9, freely available on the Internet, will also be employed. These assessments will be made at the baseline, 4-, and 12-month intervals.

The 10-item PSS [46] is a commonly used instrument to measure the extent to which one’s life is appraised as stressful. It has demonstrated adequate reliability (Cronbach alpha=.78). The PSS has been used in people with heart disease, including CHF [83]. We will use a validated French version of the PSS for this trial [84] at the baseline, 4-, and 12-month assessments.

Readiness for change in self-care will be assessed using Prochaska’s transtheoretical algorithm [47] at the baseline, 4-, and 12-month intervals. This algorithm categorizes “readiness” to make behavioral changes in one of five stages: (1) precontemplation (do not intend to make a behavioral change in the next six months), (2) contemplation (intend to start making behavioral changes within the next six months), (3) preparation (ready to start making behavioral changes within the next 30 days), (4) action (have made changes to a behavior within the last six months), and (5) maintenance (continue with the new behavior that was changed six or more months ago).

The functional capacity of the subjects will be assessed using the 6MWT [85] at the baseline and 12-month intervals. In accordance with the American Thoracic Society’s protocol, the test will use an indoor straight course of 30-40 meters and standard instructions to “walk as far as possible in six minutes.” Values will be expressed as the percent predicted value, rather than absolute distance, because the former is less susceptible to confounding factors [86].

Autonomic nervous system function of the subjects will be assessed using heart rate variability. A 10-minute recording will be collected using a three-lead ECG. The data will be sampled at 1000 Hz using LabView (version 7.1, National Instruments). A custom heart rate variability software will be used to analyze RR interval data using a fast Fourier transformation to obtain low frequency (0.04-0.15 Hz) and high frequency (0.15-0.50 Hz) spectral components. Only the Toronto sample will be asked to provide an ECG recording at baseline and 12-month intervals.

Peak aerobic power (VO_2_ peak, oxygen consumption) and the VE/VCO_2_ (rate of elimination of carbon dioxide) slope will be used to assess exercise capacity of the subjects at the baseline and 12-month intervals. The VO_2_ peak has prognostic value [87]. It is associated with the quality of life [88], and it is sensitive to change following home-based training with telehealth in CHF patients [55]. We will be collecting this information on the subjects through medical chart review.

The SHFM provides a risk estimate that has been validated among patients with CHF [49-51]. It is derived from prognostic variables (diuretic dose/kg, systolic blood pressure, percent lymphocytes, haemoglobin, etiology, ejection fraction, cholesterol, uric acid, allopurinol, serum sodium, statin, NYHA class, age, and sex) that are easily obtained from medical charts. We will review the medical charts of the subjects to extract values for these abovementioned variables at the baseline and 12-month intervals. These will then be inserted into a freely available algorithm on the Internet. The generated SHFM score will be used in our analyses.

The brain natriuretic peptide (BNP) is a neurohormone that is synthesized in and secreted from the ventricular myocardium in response to elevated ventricular wall tension and stretch, and from the activation of the sympathetic and renin-angiotensin systems. BNP is increased in patients with CHF [89], and it correlates with risk for all-cause, cardiac, and pump-failure mortality. Proinflammatory hsCRP is elevated in CHF as the disease progresses [52,54]. We will extract these data, if available, from the medical charts of subjects at the baseline and 12-month assessments.

We will also collect the anthropometric variables such as age, sex, height and weight (for body mass index), waist circumference, medications, alcohol (drinks/day), and medical history for each subject at the baseline and 12-month assessments.

### Data Collection

Data will be collected at three time points–baseline, 4-, and 12-month. Each subject will be asked to attend an in-person assessment appointment at the HF clinic from which he/she is recruited (Toronto, Montreal, or Vancouver) at each time point. During each assessment, subjects will be asked to complete specified physiological tests and self-report questionnaires (on paper or Web-based). There will be two exceptions: (1) subjects will complete the Web-based DHQ II, and (2) wear the accelerometer at home for seven days after their baseline assessment, and immediately prior to their 4- and 12-month assessments.

### Subject Compliance Monitoring

As a quality control check, we will evaluate the number of emails sent to subjects versus the number of proactive emails that subjects have opened via automated reply. This will yield a ratio of adherence to treatment (number of emails sent/number of emails opened) that will be considered as a potential covariate in supplemental outcome analyses.

Problems with adherence/compliance to preventive counseling are often due to increased response burden that is disproportionate to perceived benefit [90]. To offset this problem, we will inform subjects that they can keep the accelerometer (approximate value=US $50) as a gesture of appreciation for their participation. Transportation/parking, up to US $21, will also be reimbursed. Finally, we expect to reinforce subject motivation to comply with trial procedures by maintaining a regular schedule of e-messages over 12 months, which are likely to be perceived as being supportive in nature.

### Statistical Analysis Plan

#### Sample Size Estimation

Our sample size was not only estimated based on our own previous work, but also based on the seminal work from Heart Failure: A Controlled Trial Investigating Outcomes of Exercise Training (HF-ACTION). At the 12-month outcome in HF-ACTION [88], 53% (n=618, 95% CI 50%-56%) of subjects in exercise training had a clinically significant improvement (≥5 points) [38] from the baseline on the KCCQ [39], compared with 33% (n=386, 95% CI 30%-35%) in Usual Care. With type I error of .05 and power of .80, 93 subjects per group are required to replicate this effect. HF-ACTION was not an e-intervention, however, it utilized a 12-month home-based program to which only 40%-45% of subjects were adherent [91]. Consequently, there was only a small treatment effect for change in exercise capacity. This was correlated with KCCQ outcomes [88]-exercise group gained only 0.6 ml^.^kg^-1.^min^-1^ (interquartile range–IQR, −0.7 to 2.3) and Controls gained 0.2 ml^.^kg^-1.^min^-1^ (IQR, −1.2 to 1.4) [92]. This is likely due to low exercise intensity. CHF-CePPORT (in keeping with our previous trials of COHRT, I-START, and REACH) [23] [19,22,28] [21] is designed to improve the quality of life associated with increased self-care behaviors, including exercise. Therefore, a similar small change in exercise capacity as in HF-ACTION is expected, in association with an expected increase in the KCCQ score [39]. With alpha of .05 and power of .80, a sample of 115 subjects per group is required. Withdrawal or drop out (for any reason) has been below 6% in HF-ACTION [93] and in our previous telemonitoring trial [24]. When completion of repeated behavioral assessments are factored into subject loss, attrition was 19% in our telemonitoring trial [24] and 21% in I-START [19,22] Therefore, we conservatively plan for 21% attrition for CHF-CePPORT-final sample=278 subjects.

Our team will recruit subjects from CHF clinics in tertiary care hospitals where we hold senior positions. It is feasible to recruit 278 subjects across 3 sites: (1) Vancouver (St. Paul’s Hospital), (2) Toronto (UHN), and (3) Montreal (Montreal Heart Institute)–Years 1 and 2=248 subjects/3 hospitals/2 years=41 subjects per hospital, per year. Additionally, 30 subjects (10 per hospital) will be recruited in the first quarter of Year 3. The research clerk at the Behavioral Cardiology Research Unit, UHN, Toronto will perform coordination and monitoring of recruitment.

#### Statistical Analysis

This trial will use a 2 parallel-group design with 3 repeated assessments at baseline, 4-, and 12-months. A generalized mixed model (GMM) will evaluate the primary outcome, which is a binary code of whether the 12-month KCCQ [39] increases ≥5 points [38]. Predictors will include baseline KCCQ, age, sex, body mass index, and Group (e-Counseling vs Control). The primary outcome will be coded as a “failure” in the event of CHF hospitalization or mortality, but not for “elective” medical procedures (eg, cardiac resynchronization therapy). GMM with repeated measures will assess binary secondary outcomes across 4- and 12-month intervals (eg, KCCQ and smoking status). This analysis is optimal as it adjusts for serial correlations across repeated measures and between individual subjects in each group. Predictors will include baseline KCCQ, age, sex, body mass index, time (4- vs 12- month assessment interval), and Group (e-Counseling vs Control). Significant interactions or main effects will be followed by Bonferroni post hoc tests for significance, *P*<.05, 2-sided. Data missing at random will be handled by multiple imputations. For continuous secondary outcomes, linear mixed models (LMM) [94] for repeated measures with a random intercept will assess whether within-subject improvement across 4- and 12-month intervals is independently associated with our e-Counseling protocol. This analysis adjusts for serial correlations across repeated measures and between subjects in each Group. Predictors will include the baseline dependent variable, age, sex, body mass index, time (4- vs 12-month assessment interval), and Group (e-Counseling vs Control). LMM with a random intercept will also evaluate whether e-Counseling vs Control demonstrates improvement at the 12-month interval in VO_2_ peak, NT-proBNP, hsCRP, and the SHFM.

#### Planned Subgroup Analysis

Subanalyses will explore whether therapeutic changes in primary or secondary outcomes following e-Counseling differ significantly within subgroups–sex, age, and income level. We will use GMM to evaluate the dose-response relationship between e-Counseling and improvement in our primary outcome; and LMM to evaluate this dose-response relationship for prognostic measures of CHF at the 12-month outcome using a ratio of adherence to treatment (number of emails sent/number of emails opened).

#### Quality Control and Quality Assurance Procedures

Three committees will be established for quality control and quality assurance purposes. The Steering Committee will hold teleconference meetings every 3 months to review trial progress, overall outcome rates, issues related to evaluation of primary or secondary outcomes, and response of research staff to any adverse incidents. This group will recommend whether our trial should continue without protocol modification, with modification, or whether it should be terminated. The Outcome Adjudication Committee will meet every 6 months by teleconference to adjudicate issues related to primary outcome status of subjects. The Safety and Monitoring Committee will meet annually with the option of expedited meetings in the event of an urgent issue or unexpected “serious adverse event.”

## Discussion

### CHF-CePPORT Benefits to CHF Patients

It is critical for preventive eHealth care in Canada to establish a foundation upon which a pan-Canadian e-platform can be built for patients with CHF in order to improve the quality of life and adherence to self-care behaviors. It is reasonable to expect that CHF-CePPORT will provide data that is indispensible in helping investigators in Canada to develop a compelling Phase 3 trial where the independent benefit of e-Counseling for CHF can be evaluated with regard to decreasing HF hospitalizations and mortality-as has been shown for telehealth [35,70,95].

### CHF-CePPORT Findings

Findings from CHF-CePPORT will also help guide the development of CHF e-Counseling services provided by the Heart and Stroke Foundation of Canada. Their Consumer eHealth platform offers visibility and accessibility for disseminating information and resources developed by CHF-CePPORT through its website. In 2011, this e-platform accommodated 395,044 users who searched for heart health information, while 160,600 users completed structured risk assessments. Second, our results will be submitted for presentation at national meetings and peer-reviewed publications. Third, the Heart and Stroke Clinical Update is an annual continuing medical education conference hosted by the Heart and Stroke Foundation of Canada for primary care physicians. Pending the results of this study, there is interest to develop a workshop for Canadian physicians and health professionals on the use of our e-platform to promote self-care in patients with CHF. As results become available, our team will develop a knowledge translation supplement grant.

### The e-Platform in CHF-CePPORT

The proposed e-platform in CHF-CePPORT is designed to complement (rather than compete with) e-programs that are housed in collaborating institutions. Following our trial, our aim is to build supplementary e-links to specialized programs in participating CHF clinics within Canada. At the same time, we expect that the e-platform in CHF-CePPORT will evolve as an e-Counseling resource for CHF clinics through collaborations with other investigators. To that end, members of our research team are affiliated with societies that are engaged in knowledge dissemination and application. These affiliations will permit direct dissemination of study outcomes to key opinion leaders and facilitate uptake of new knowledge to a broader audience.

## Abbreviations

6MWT: 6-minute walk test
BNP: brain natriuretic peptide
CHF: chronic heart failure
CHF-CePPORT: Canadian e-Platform to Promote Behavioral Self-Management in Chronic Heart Failure
COHRT: Community Outreach Heart Health and Risk Reduction Trial
CONSORT: Consolidated Standards of Reporting Trials
DHQ II: Diet History Questionnaire II
ECG: electrocardiography
GAD-7: generalized anxiety disorder
GMM: generalized mixed model
HF-ACTION: Heart Failure: A Controlled Trial Investigating Outcomes of Exercise Training
hsCRP: high sensitivity C-reactive protein
IQR: interquartile range
I-START: Internet-Based Strategic Transdisciplinary Approach to Risk Reduction and Treatment
KCCQ: Kansas City Cardiomyopathy Questionnaire
LMM: linear mixed models
LVEF: left ventricular ejection fraction
MI: motivational interviewing
MPR: medication possession ratio
NT-proBNP: N-terminal pro-brain natriuretic peptide
NYHA: New York Heart Association
PASE: Physical Activity Scale for the Elderly
PHQ-9: Patient Health Questionnaire
PSS: Perceived Stress Scale
REACH: Promotes Blood Pressure Reduction and Therapeutic Lifestyle Change in Hypertension
RR: relative risk
SHFM: Seattle Heart Failure Model
UHN: University Health Network
